# Apoptotic vesicles: emerging concepts and research progress in physiology and therapy

**DOI:** 10.1093/lifemedi/lnad013

**Published:** 2023-04-17

**Authors:** Yu Fu, Yifan He, Di Wu, Bingdong Sui, Yan Jin, Xuefeng Hu, Songtao Shi

**Affiliations:** Hospital of Stomatology, Guanghua School of Stomatology, Sun Yat-sen University, South China Center of Craniofacial Stem Cell Research, Guangdong Provincial Key Laboratory of Stomatology, Guangzhou 510055, China; Fujian Key Laboratory of Developmental and Neural Biology and Southern Center for Biomedical Research, College of Life Sciences, Fujian Normal University, Fuzhou 350117, China; Hospital of Stomatology, Guanghua School of Stomatology, Sun Yat-sen University, South China Center of Craniofacial Stem Cell Research, Guangdong Provincial Key Laboratory of Stomatology, Guangzhou 510055, China; Hospital of Stomatology, Guanghua School of Stomatology, Sun Yat-sen University, South China Center of Craniofacial Stem Cell Research, Guangdong Provincial Key Laboratory of Stomatology, Guangzhou 510055, China; Research and Development Center for Tissue Engineering, The Fourth Military Medical University, Xi’an 710032, China; Research and Development Center for Tissue Engineering, The Fourth Military Medical University, Xi’an 710032, China; Fujian Key Laboratory of Developmental and Neural Biology and Southern Center for Biomedical Research, College of Life Sciences, Fujian Normal University, Fuzhou 350117, China; Hospital of Stomatology, Guanghua School of Stomatology, Sun Yat-sen University, South China Center of Craniofacial Stem Cell Research, Guangdong Provincial Key Laboratory of Stomatology, Guangzhou 510055, China

**Keywords:** extracellular vesicles, apoptosis, apoptotic vesicles, physiological homeostasis, regenerative therapeutics

## Abstract

Apoptosis represents the dominant form of programmed cell death and plays critical roles in maintaining tissue and organ homeostasis. A notable population of extracellular vesicles (EVs) is generated during apoptosis, known as apoptotic vesicles (apoVs). These apoVs are increasingly the subject of studies concerning their identity and mechanisms of production, which have been revealed unique biological and functional characteristics that are emerging as crucial regulators for diverse processes. Furthermore, apoVs have been gradually noticed for their essential role in regulating the physiology of various organ systems *in vivo*, and growing evidence suggests that apoV dysregulation contributes to age- and pathology-associated tissue alterations. Importantly, apoVs can be therapeutically harnessed to unleash their potential in treating several diseases such as immune disorders, osteoporosis, cutaneous wound and acute liver failure; these vesicles, mainly derived from cultured mesenchymal stem cells, hold great translational promise. Here we review the current landscape of scientific knowledge about apoVs, with emphasis on mechanistic insights into how apoVs contribute to organismal health and disease, which also provide novel cell-free strategies for EV-based regenerative therapeutics.

## Introduction

Programmed cell death (PCD) refers to cellular suicide pathways that promote the removal of stressed, damaged, malignant, or infected cells. PCD in its various forms, including apoptosis, autophagy, pyroptosis, ferroptosis, plays a fundamental role in animal development and pathogenesis as well as physiological homeostasis [1–5]. As the most prominent mode of PCD, apoptosis has been known as a caspase-dependent process, which is regulated by extrinsic ligand-receptor integration and intrinsic genetic mechanisms [6, 7]. Importantly, apoptosis contrasts with necrosis, in which dead cells are cleared by phagocytosis without release of immunogenic intracellular materials to trigger immune reaction and thereby maintain tissue and organ homeostasis [8, 9]. It has been widely reported that over 50 billion cells undergo apoptosis and generate a large number of apoptotic vesicles in the human body every day [5, 10–12]. Nevertheless, the biological properties and physiological functions of these apoptotic products remain to be elucidated.

Extracellular vesicles (EVs) consist of cellular materials in a lipid bilayer and are shed from the living cell membrane or *via* exocytosis of endosomal membrane [13, 14]. Depending on their cellular origin, EVs are capable of encapsulating various cellular components, including proteins, DNAs, RNAs, lipids, and metabolites [15–17]. EVs exhibit extensive biological effects *via* surface molecules that target recipient cells under specific physiological and pathological contexts, inducing signal transduction cascades based on receptor-ligand interaction or being internalized by endocytosis to deliver bioactive content [18–21].

Recently, a series of studies have revealed that cells undergoing apoptosis can produce heterogeneous apoptotic vesicles (apoVs), which exhibit molecular characteristics quite distinct from exosomes and microvesicles (Table 1) [16, 22–26]. Furthermore, it has been demonstrated that apoVs are specific apoptotic products with extensively involvement in the maintenance of organ/tissue homeostasis and in disease development based on paracrine action [23]. Notably, systemic infusion of exogenous mesenchymal stem cell (MSC)-derived apoVs presents unique therapeutic effects in various diseases, such as immune disorders, osteoporosis, skin injuries, and tumors [25]. Importantly, accumulating evidence showed that MSC-derived apoVs possess advantages in low immunogenicity, easy storage, and amenability to engineering for drug delivery [27–29]. However, the means by which apoVs are produced and the mechanisms underlying their physiological and therapeutic effects remain elusive. Here we review cutting-edge knowledge regarding the biogenesis, physiological characteristics, and pre-clinical application of apoVs.

**Table 1.** Comparison of apoVs with exosomes and microvesicles

	Exosomes	Microvesicles	apoVs
Common markers	CD9, CD63, CD81, Flotillin, TSG101, ceramide, and Alix	Annexin A1	C1q, PS, Fas, Intergrin alpha-5, Syntaxin-4, Caveolin-1, Cavin1
Cellular components	Proteins MHCI and MHCII Lipid rafts Targeting and adhesion proteins mRNAs miRNAs circRNAs lncRNAs	Proteins MHCI and MHCII Lipid rafts Targeting and adhesion proteins mRNAs miRNAs circRNAs lncRNAs	Proteins DNAs RNAs Lipids and metabolites Nuclear substances
Size and origin	30–100 nm in diameter; Form in multivesicular bodies	50–1000 nm in diameter; Form in plasma membrane	100–1000 nm in diameter; Form in plasma membrane, multivesicular bodies or apoptopodia
Potential applications	Metabolic diseases, cardiovascular diseases, reproductive diseases	Cancer, cardiometabolic diseases, neurologic diseases, infectious diseases	Immune disorders, osteoporosis, skin injuries, multiple myeloma, lethal acute liver failure, diabetes

## Apoptosis and biogenesis of the vesicular products

**Figure 1.**
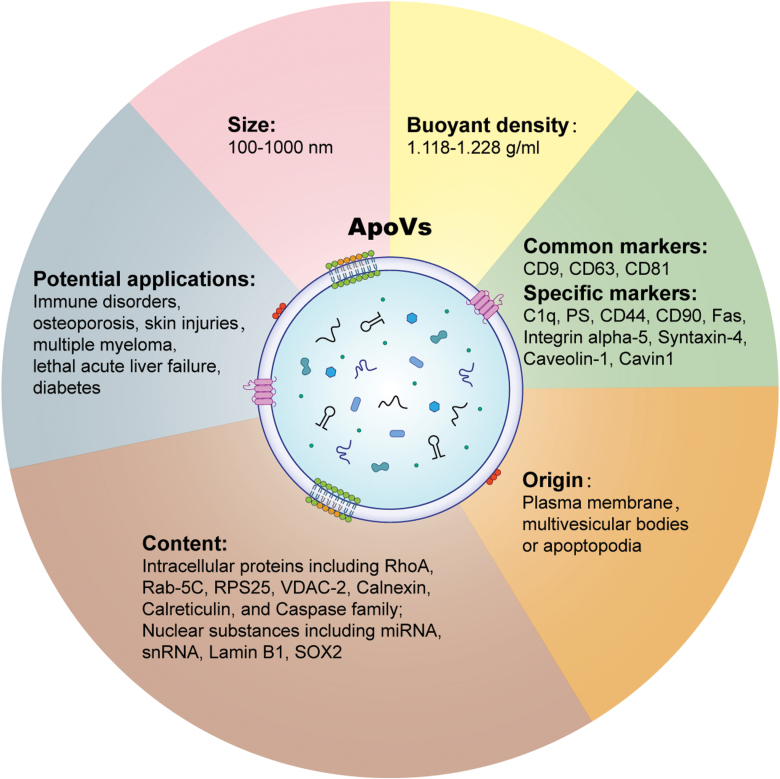
**Characterization of apoVs.**Descriptions of apoVs have relied on size and defined them as having a diameter of between 100–1000 nm, as well as buoyant density between 1.118–1.228 g/mL. ApoVs have common markers including CD9, CD63, and CD81, and also possess ubiquitous apoptotic markers phosphatidylserine (PS) and C1q as well as parental cell-derived markers such as mesenchymal stem cell (MSC) positive marker CD44 and CD90. ApoVs are released *via* direct membrane budding or formed in intracellular multivesicular bodies (MVBs) through the invagination of plasma membrane and endosomal membrane. Specifically, apoVs can be generated based on PANX1-dependent mechanisms involving a previously unrecognized type of membrane protrusion that resembles a “bead-on-a-string” structure, termed apoptopodia. The content of apoVs mainly includes lipids, specific proteins, and nuclear substances. To date, apoV transplantation has been applied in a variety of diseases and conditions including immune disorders, osteoporosis, skin injuries, *etc*.

Apoptosis refers to the spontaneous and orderly cell death that serves to remove unwanted cells, contributing to organismic development, tissue and organ homeostasis, aging, and pathogenesis [5, 30–34]. Accumulating studies have characterized the morphological changes of apoptotic cells, such as shrinkage, membrane blebbing, and nuclear condensation [1, 22, 35–37]. It has long been known that apoptotic cells generate apoptotic bodies (apoBDs), but it has only recently been revealed that smaller apoVs are also existed [22]. Further studies have demonstrated that these two kinds of vesicle-like bodies are heterogeneous, with completely different sizes, cargo, and production mechanisms [24, 38]. The formation of apoBDs occurs after active caspases cleaving rho-associated coiled-coil-containing protein kinases 1 (ROCK1), which phosphorylates and activates blebbing-regulating myosin light-chain (MLC) and the caspase-cleaved membrane receptor plexin B2 (PLXB2), finally inducing cell contractility and forming membrane blebs on the cell surface [39]. The dying cell’s membrane then gradually protrudes and produces apoBDs [40]. It is currently known that apoVs can be produced in a variety of unique modes according to their cells of origin and physiopathological contexts, resulting in production of heterogeneous apoV subtypes [23]. On the one hand, some apoVs are released through direct membrane budding during the progression of apoptosis [41, 42]. On the other hand, the biogenesis of apoVs is similar to that of EVs from living cells, which are first formed in intracellular multivesicular bodies (MVBs) through the invagination of plasma membrane and endosomal membrane, but apoVs are specifically characterized by apoptosis for final secretion [14, 43]. Intriguingly, the process of apoV release is triggered by cellular sphingosine1-phosphate (S1PR)-coupled Gβγ stimulating the actin cytoskeleton, a process which is also controlled by the apoptotic signaling molecule caspase-3 [43]. Notably, a caspase-activated pannexin 1 channel (PANX1) recently has been revealed to be involved in novel apoV formation, termed apoptopodia [22, 44]. Apoptopodia has been reported to generate a previously unknown type of membrane protrusion that resembles a “beads-on-a-string” structure in monocytes through PANX1-dependent mechanisms [45]. To summarize, based on the diverse progression mechanisms, cell origins, and physiological and pathological contexts of apoBDs and apoVs, different subtypes of these apoptotic products have been discovered and identified, while the molecular characteristics and biological functions of these subtypes still remain to be investigated (Fig. 1).

## Biological and physiological characteristics of apoVs

With the growing development of EV analytical techniques, several studies have revealed the phenotypic and functional properties of apoVs [46–48]. Previous studies have characterized EVs into three main types by size and biogenetic pathway: exosomes (30–100 nm), microvesicles (50–1000 nm), and apoBDs (1000–5000 nm) [14, 49]. The size and potential of apoVs, which diverge from those of EVs, have recently been determined by nanoparticle tracking analysis (NTA), which found a size range from 100 to 1000 nm and mean membrane potential of approximately −28 mV (Table 1) [38]. Furthermore, it has been reported that the buoyant density of apoVs is 1.118–1.228 g/mL, denser than exosomes and microvesicles, which range from 1.05–1.15 g/mL and 1.03–1.10 g/mL, respectively [16, 38]. Emerging techniques based on bioinformatics have also been used to screen, predict, and analyze potential novel cellular contents of apoVs. Liquid chromatography followed by tandem mass spectrometry (LC–MS/MS) analysis has shown that EV content mainly includes proteins related to extracellular matrix, endoplasmic reticulum, immune response, and cell adhesion functions, but apoVs are enriched with numerous functional proteins related to cellular behavior and metabolism, transport, and regulation of various diseases [38, 50, 51]. In addition, the membrane molecular components of apoVs not only have common markers of EVs, such as CD9, CD63, and CD81, but also possess the ubiquitous apoptotic markers phosphatidylserine (PS) and C1q [27, 29, 52]. Notably, apoVs can inherit their parental cells’ properties, which are considered to contribute to their diversity and heterogeneity. As one notable example, MSC-derived apoVs highly express the positive MSC markers CD29, CD44, and CD90 but are negative for expression of CD34 and CD45 [38]. Pluripotent stem cell (PSC)-derived apoVs have also been shown to carry the pluripotent-specific molecule SOX2 in a caspase 3-dependent manner [53]. As for the intracellular contents, multiple reaction monitoring (MRM) combined with western blot analysis revealed that MSC-derived apoVs specifically package 13 proteins, including Fas, Integrin alpha-5, Syntaxin-4, CD44, RhoA, Caveolin-1, Cavin1, Rab-5C, RPS25, Lamin B1, VDAC-2, Calnexin, and Calreticulin [38]. Small RNA profiling results revealed that the transcriptome of osteoclast-derived apoVs shows strong similarities to the corresponding parental cells, specifically related to endothelial progenitor cell proliferation and differentiation [54]. Under pathological conditions, glioblastoma-derived apoVs inherit the splicing factor RNA-binding motif protein 11 (RBM11), which could be internalized in recipient cells to switch splicing of murine double minute 4 (MDM4) and Cyclin D1, triggering oncogene expression [55]. Taken together, these findings suggest that apoVs have unique biomarkers and cellular contents compared to apoBDs and EVs, contributing to their unique targeting and functional modulation in specific physiological and pathological contexts. The physical properties and the molecular contents of apoVs contribute to their biological functions and are completely distinct from those of apoBDs and EVs, thus apoVs clearly represent a previously unappreciated type of apoptotic products. However, the defining characteristics of apoVs used in the field are still blurry because of their multitudinous tissue origins *in vivo* and induction methods *in vitro* (Table 2) [23]. Accordingly, we suggest that it will be important for clarity in future research to standardize terms for diverse subtypes of apoVs in the light of their sizes, induction methods, specific cellular contents, and biological effects.

**Table 2.** Characteristics and function of apoVs

Origin	Inducing method	Centrifugal force	Size	Functional molecule	Biological effects	Terms used	References
Mice B16 cell line *in vitro*	Doxorubicin	25,000 *g*	357 ± 112.1 nm	Fibrin	Involving procoagulant activity and the anti-cancer response	Apoptotic vesicles	[56]
Mice *in vivo*	*M. tuberculosis*	1800 *g*	/	Mycobacterial antigens	Activating CD8 T cells	Apoptotic blebs	[57]
Human lymphoblasts *in vitro*	UV-B irradiation	100,000 *g*	100 nm–1 μm	MHC II	Suppressing immune responses	Apoptotic blebs	[58]
Mice bone marrow MSCs *in vitro*	Staurosporine	16,000 *g*	100–350 nm, averaging 200 nm	Phosphatidylserine	Preventing Th17 differentiation and memory formation; Ameliorating inflammation and joint erosion in murine arthritis	Apoptotic vesicles	[59]
Human bone marrow MSCs *in vitro*	Staurosporine	16,000 *g*	Less than 1 μm	Calreticulin	Restoring liver macrophage homeostasis and ameliorating T2D	Apoptotic vesicles	[50]
RAW264.7 macrophages *in vitro*	Staurosporine	16,000 *g*	240.6 ± 115 nm	miR-155	Transporting miRNAs and regulating multidirectional differentiation of stem cells	Apoptotic vesicles	[60]
Human hepatocyte *in vivo*	Hepatectomy	1000 *g*	2.94 ± 0.03 μm	/	Contributing to liver regeneration	Apoptotic extracellular vesicles	[61]
Mice and MLO-Y4 osteocyte cell line *in vitro*	Serum-starvation	20,000 *g*	20 nm–1 μm	/	Triggering osteoclastogenesis and localizing bone destruction	Apoptotic bodies	[62]
Bone marrow macrophages, preosteoclasts, and mature osteoclasts *in vitro*	Staurosporine	3000 *g*	1–4 μm	Similar genetic phenotype with the corresponding parental cells/non-coding RNAs	Contributing to bone remodelling	Apoptotic bodies	[54]
Mature osteoclasts *in vitro*	Alendronate	3500 *g*	1–4 μm	Vesicular RANK	Contributing to bone remodeling/osteogenic differentiation	Apoptotic bodies	[63]
Human Glioblastoma cells *in vitro*	γ irradiation/temozolomide/cisplatin	120,000 *g*	50–200 nm	Spliceosomal proteins/snRNAs/RBM11	Promoting malignancy of glioblastoma	Apoptotic extracellular vesicles	[55]
Mice B16-F1 or B16-OVA cells	Doxorubicin	100,000 *g*	103–816 nm/220–531 nm	Fibrin/Thrombin/PMEL	Involving procoagulant/cancer-related thrombosis/anti-cancer response	Apoptotic blebs or vesicles	[56]
Human glycan-modified melanoma cell line Mel-JuSo	Bortezomib	10,000 *g*	100–1000 nm	Glycan	Antigen source for antitumor vaccination	Apoptotic extracellular vesicles	[64]
Mice bone marrow MSCs *in vitro*	Staurosporine	2000 *g*	1000–5000 nm	Ubiquitin ligase RNF146/miR-328-3p/Wnt	Contributing to MSC and bone homeostasis maintenance	Apoptotic bodies	[27]
Human bone marrow MSCs *in vitro*	H_2_O_2_	/	/	TSG-6	Preventing Scar formation and immunomodulation	Apoptotic hMSCs	[65]
Human MSCs *in vitro*	Recipient cytotoxic cells	/	/	Indoleamine 2,3-dioxygenase	Contributing to immunosuppression.	Apoptotic MSCs	[66]
Human bone marrow or adipose MSCs; Mice bone marrow MSCs	Staurosporine H_2_O_2_/starvation	16,000 *g*	100–1000 nm	Fas	Activating platelet	Apoptotic vesicles	[38]
Mice thymocytes; Human Jurkat cells	Gamma cell 40 irradiator or UV irradiation	10,000 *g* or 180,000 *g*	50–200 nm	Phosphatidylserine/FOXO3	Contributing to TGF-β production in macrophages/anti-inflammation	Apoptotic cell-derived EVs	[67]
Mice bone marrow MSCs	Staurosporine	16,000 *g*	100–1000 nm	AMPK/SIRT1/NF-κB pathway	Inhibiting inflammatory macrophages and osteoclast formation	Apoptotic extracellular vesicles	[68]
Mice bone marrow MSCs, T cells, and NIH/3T3 cell line; Human/umbilical cord MSCs	Staurosporine/ ultralow-power light irradiation/BH3-mimetic ABT-737/ CD95/Fas antibody	16,000 *g*	100–800 nm	VAMP3	Restoring hepatic ploidy homeostasis	Apoptotic extracellular vesicles	[28]
Human bone marrow MSCs	Staurosporine	16,000 *g*	150.1 ± 14.8 nm	Fas ligand	Improving electrostatic charge-directed target/ neutrophil apoptosis	Apoptotic vesicles	[69]
Mice bone marrow MSCs	Staurosporine	16,000 *g*	50–250 nm	Fas ligand	Inducing multiple myeloma cell apoptosis	Apoptotic extracellular vesicles	[70]
Mice bone marrow MSCs	Staurosporine	16,000 *g*	179.1 ± 2.4 nm	Rab7	Contributing to autolysosome formation restoration/bone homeostasis maintenance	Apoptotic vesicles	[71]
Mice MSCs	Staurosporine	16,000 *g*	50–200 nm	Wnt	Contributing to skin and hair homeostasis maintenance	Apoptotic extracellular vesicles	[72]
Human deciduous pulp stem cells	Staurosporine	16,000 *g*	100–800 nm	Mitochondrial Tu translation elongation factor	Involving angiogenesis/dental pulp regeneration	Apoptotic vesicles	[73]
Human ESCs, iPSCs, and umbilical cord MSCs	Staurosporine	16,000 *g*	106 ± 2.9 nm	SOX2/Hippo signalling pathway	Contributing to skin wound healing	Apoptotic vesicles	[53]

## ApoV in regulation of various organ systems

Understanding the regulatory landscape of apoV behaviors is necessary to decipher their roles in tissue and organ homeostasis. As is traditionally known about apoptosis, apoVs possess immune regulatory effects through mediating apoptotic cell clearance and intercellular communication [23, 74]. Apoptotic deficiency has been demonstrated as a key player in autoimmune disease, in which cells that are unable to undergo apoptosis release their immunogenic intracellular materials [32, 75, 76]. Furthermore, accumulating studies have suggested that apoVs possess a significant immunomodulatory capacity similar to that of EVs through aiding antigen presentation; e.g., dendritic cell-derived apoVs carry major histocompatibility complex (MHC) II to activate CD4^+^ T cells [56, 57, 77]. It is notable that antigen-presenting capabilities of apoVs are also modulated by the engulfment of dendritic cells, suppressing immune responses by downregulating MHC II molecules [58]. Wang et al. [59] recently revealed that MSC-derived apoVs directly interact with CD4^+^ T cells and inhibit CD25 expression and interleukin (IL)-2 production mediated by exposed PS. With regard to phagocytosis of macrophages, apoVs promote macrophage reprogramming in an efferocytosis-dependent manner, contributing to inhibition of macrophage accumulation and inducing polarization of macrophages towards an anti-inflammatory phenotype [50]. Moreover, macrophage-derived apoVs have been indicated to suppress osteogenesis and promote adipogenesis of MSCs *via* delivering microRNA155 (miR155) to regulate Smad2 signaling [60].

Over 50 billion cells produce apoVs each day in the human body to maintain tissue and organ homeostasis, which is crucial for development and normal tissue turnover [10, 78, 79]. Early studies have reported that apoptosis of nerve cells during the development of nervous system is necessary, as it sculpts the developing brain; conversely, it plays a potentially key role in neurodegenerative diseases [80, 81]. In the development of the germ line, apoptosis is a significant physiological process for modulating the size of the male or female sex gland, which directly controls the quality of germ cells and in turn plays a major role in determining fertility [82]. The delicate equilibrium between apoptosis and compensatory proliferation also plays an essential role in animal development. In the *Drosophila* wing imaginal disc, compensatory proliferation induced by apoptotic cells is involved in the developmental potential of the affected tissue, triggering Hedgehog (Hh) signaling activation in differentiating eyes [83]. Apart from the finely coordinated process of development, studies have also investigated apoV-specific regulatory mechanisms in homeostatic maintenance of various tissues. Recently, untargeted metabolomic results showed that apoptotic brown adipose tissue releases a specific pattern of metabolites, particularly inosine, as a “replace me” signal that contributes to thermogenic fat modulation and counteracts obesity [84]. Brandel et al. [61] indicated that hepatectomy-induced apoVs act as a trigger that stimulates neutrophils to adopt a non-inflammatory, regenerative phenotype that may contribute to liver regeneration. Further exciting liver-apoV metabolic research revealed that apoVs undergo specialized internalization by hepatocytes (HCs) based on a sugar recognition system and safeguard diploid HCs by assembly of the ApoV–Golgi Complex (AGC) [28]. Gupta et al. [85] discovered CRK-containing apoVs in damaged mouse glomeruli, which might have regenerative effects in the kidney. As previously reported, recipient-impaired bone marrow MSCs (BMMSCs) can engulf apoVs, which reuse ubiquitin ligase RING finger protein 146 (RNF146) and miR-328-3p to promote endogenous BMMSC recovery [27]. In this regard, osteocyte-derived apoVs have been shown to trigger osteoclastogenesis and induce localized bone destruction [62]. Intriguingly, studies indicated that osteoclast-derived apoVs promote osteogenic differentiation in bone remodeling through receptor activator of nuclear factor kappa-Β ligand (RANKL)-mediated reverse signaling [54, 63]. Recent apoV research has made significant progress in elucidating their roles in the skin and the integument, which is an essential metabolic system involved in endogenous apoV migration and clearance, through studies using a parabiotic mouse model to examinate circulatory factors transferring from one animal to another [72]. Mechanistically, it has been further revealed that treadmill exercise increased the number of green fluorescent protein (GFP)-positive apoVs migrating to the skin. But tail suspension decreased the number of GFP-positive apoVs migrating to the skin. The expression of Wnt signaling inhibitor Dickkopf-1 (DKK1) in the circulation was inhibited in the treadmill group but increased in the tail suspension group [72]. However, whether mechanical force mediates apoV migration by Wnt signaling regulation still remains to be elucidated.

Inducing tumor cell apoptosis has long been a crucial concept in cancer treatment, so it is therefore important to consider how tumor cell-derived apoVs are involved in tumorigenesis [86]. On the one hand, apoptotic glioblastoma cells facilitate proliferation and therapeutic resistance of tumors by secreting apoVs with splice factor RBM11 to affect RNA splicing in recipient cells [55]. On the other hand, it has been revealed that apoVs envelop the tumor-associated antigen premelanosome protein (PMEL) and contribute to the transport of tumor antigens to antigen-presenting cells, promoting robust antitumor immune response [56]. It has also been documented that tumor-derived apoVs are an abundant source of tumor-associated antigens (TAAs), which can efficiently deliver antigens *via* manipulating the expression of DC-targeting ligands [64]. Intriguingly, normal cell-derived apoVs can induce multiple myeloma (MM) cell apoptosis and inhibit MM cell growth, using Fas ligand (FasL) to induce MM cell apoptosis *via* Fas pathway [70]. In brief summary, apoVs derived from diverse physiopathological organs are heterogeneous and contribute to diverse biological functions which are extensively involved in immunomodulation, tissue and organ homeostasis maintenance, and disease development. Nevertheless, the regulatory processes of apoV production still remain elusive, and deciphering the biogenesis mechanisms of apoVs corresponding to their organ-specific properties might shape our understanding of apoV function *in vivo*.

## Harnessing apoVs for tissue regeneration and disease therapy

**Figure 2.**
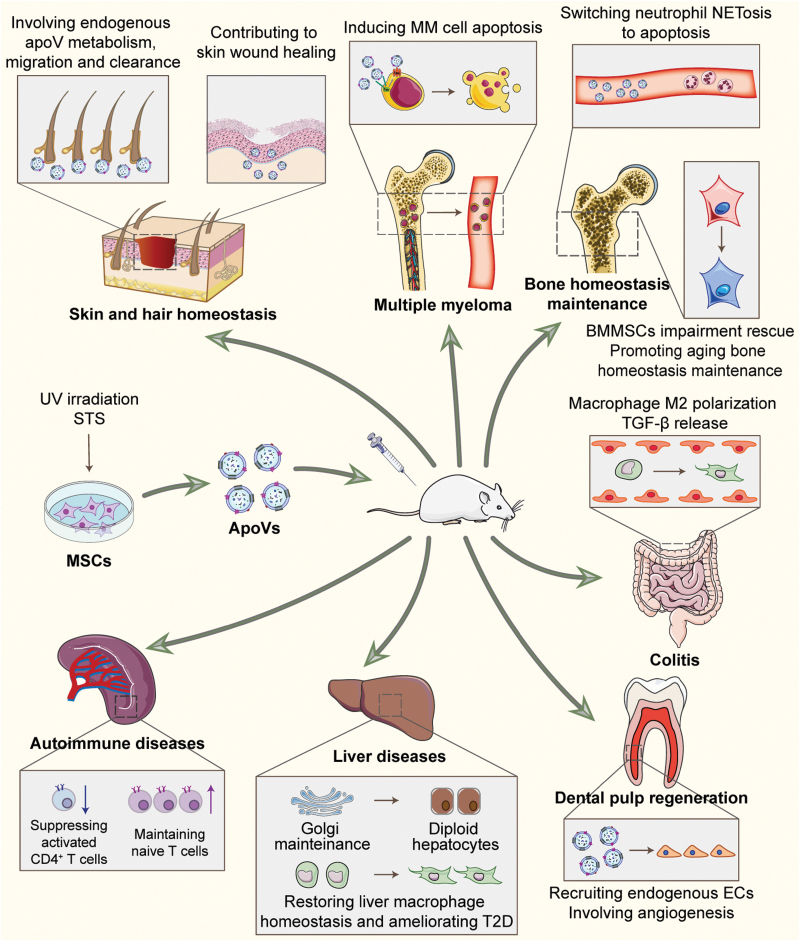
**The therapeutic effects of apoVs in various diseases.** MSCs in culture can be induced to generate apoVs under the stimulus of staurosporine (STS) or UV irradiation, and the apoVs can then be collected and infused systematically into circulation. ApoVs carry bioactive contents to recipient cells to regulate tissue homeostasis and immune response. Briefly, regarding modulation of immune systems, apoV treatment can stimulate macrophages polarization to ameliorate colitis and type 2 diabetes (T2D). ApoVs can effectively regulate CD4^+^ T cells and switch neutrophil NETosis to apoptosis for immunomodulation. Furthermore, apoVs prolong the lifespan of multiple myeloma (MM) mice through inducing MM cell apoptosis. The skin and the integument, an essential metabolic system, contribute to apoV migration and clearance. With regard to tissue regeneration, apoVs can be engulfed by bone marrow MSCs (BMMSCs) to recover BMMSC function, which remarkably rescues osteoporosis. Human deciduous pulp stem cells (hDPSCs)-derived apoVs inherit a specific angiogenic property from their parent cells that facilitates angiogenesis and dental pulp regeneration. MSC-derived apoV infusion also efficiently protects mice from lethal acute liver failure by promoting liver regeneration through safeguarding diploid hepatocytes (HCs).

In view of apoVs’ systemic regulation capacity in tissue and organ homeostasis, it is natural to wonder whether exogenous apoVs possess unique therapeutic effects. Indeed, numerous studies of the properties and functions of apoVs have focused on MSC-induced apoptosis *in vitro* [25, 87]. MSCs are non-hematopoietic stromal cells that possess clonogenic properties with multilineage differentiation capabilities *in vitro*, which have been discovered in a variety of tissues including the adipose tissue, umbilical cord, and tendons, as well as in the orofacial region [88–95]. To date, inspired by MSCs’ potent self-renewal and immunoregulatory properties *in vivo*, MSC transplantation (MSCT) has been extensively applied in pre-clinical and clinical trials aimed at tissue regeneration and immunological disease therapy [96–99]. Traditionally, the mechanisms by which MSCs exert therapeutic effects are considered to be direct effects of engraftment and differentiation of recipient tissue alongside indirect effects of paracrine mechanisms including cytokine and EV secretion [99–101]. However, recent studies have shown that exogenous MSCs undergo apoptosis after infusion, which is hypothesized to be a novel mechanism exerting indispensable therapeutic effects in MSCT [27, 65, 66]. Interestingly, MSC infusion also induces Fas/FasL pathway-mediated recipient T cell apoptosis, after which the apoptotic T cells are phagocytosed by macrophages and upregulate Tregs for immune balance [102]. Accordingly, from a translational point of view, detailed in-depth dissection of the role of apoptosis in MSCT will add to the prevailing knowledge for pre-clinical and clinical application. To date, accumulating evidence has documented that MSC-derived apoV transplantation plays an important role in tissue and organ homeostasis maintenance and immunomodulation in a variety of diseases (Fig. 2) [25].

As mentioned above, apoVs carry parental MSC-specific biomolecules, and also inherit MSCs’ potent tissue regeneration and immunoregulation properties [38]. With regard to modulation of immune systems, apoV treatment has been claimed to stimulate macrophages to upregulate transforming growth factor-β (TGF-β) production, triggering suppression of colitis in an experimentally induced rodent model [67]. A recent study has also indicated that MSC-derived apoVs inhibit proinflammatory macrophages *via* AMPK/SIRT1/NF-κB pathway, alleviating *Porphyromonas gingivalis*-LPS (Pg-LPS)-induced inflammation [68]. Furthermore, MSC-derived apoV infusion promotes transformation of macrophages to an anti-inflammatory phenotype, restoring liver macrophage homeostasis and counteracting type 2 diabetes (T2D) [50]. MSC-derived apoV infusion also efficiently protects mice from lethal acute liver failure by promoting liver regeneration [28]. Wang et al. [59] discovered that MSC-derived apoVs effectively ameliorate lupus and arthritis in an apoptotic deficiency model by regulating CD4^+^ T cells. It is also notable that MSC-derived apoVs can attenuate sepsis through switching neutrophils NETosis and interacting with recipient cells *via* apoptosis Fas/FasL signaling pathway [69]. As an antitumor therapy, systemic infusion of apoVs prolongs the lifespan of MM mice, an effect which also depends on regulating Fas/FasL signaling pathway in MM cells [70].

Exogenous MSC-derived apoV infusion has also achieved great advances in tissue regeneration and wound repair. Delivery of apoVs remarkably rescues osteoporosis by transferring miR-328-3p and ubiquitin ligase RNF146 to recipient BMMSCs, activating canonical Wnt signaling to induce endogenous BMMSC functional recovery [27]. Notably, apoVs derived from younger MSCs can rejuvenate aging BMMSCs through restoring autolysosome formation, providing a potential approach for treating age-related bone loss [71]. As for skin repair and regeneration, it has been revealed that exogenous apoVs activate Wnt signaling pathway in skin and hair follicle MSCs, which promotes wound healing and hair growth [72]. Intriguingly, stem cells in early stages, such as embryonic stem cells and induced pluripotent stem cell (iPSC)-derived apoVs are more effective at accelerating cutaneous wound healing through transferring SOX2 to skin MSCs [53]. Moreover, the specific angiogenic property of human deciduous pulp stem cells (hDPSCs) is well known, and treatment with apoVs derived from hDPSCs facilitates angiogenesis and dental pulp regeneration [73]. As mentioned above, MSC-derived apoV treatment has achieved *de novo* restoration of multiple tissue injuries and immune diseases, and further work should be dedicated to launch finely tuned therapeutic trials to confirm the suitability and efficacy of apoVs derived from different tissues, age stages, and types of cells. Taken together, the pre-clinical trials conducted thus far have established the recognition of MSC-derived apoV therapeutic effects based on physiological context and developmental origin, representing an indispensable basis for future clinical translational medicine.

## Conclusions and perspectives

The last decade has witnessed increasing understanding of how apoptosis and apoptotic products mediate physiological homeostasis maintenance and disease development [103–106]. Extensive research results have identified a novel class of apoptosis-induced vesicle-like bodies called apoVs, which can be distinguished from previously defined apoBDs through their unique physical properties, biological markers, and contents [38]. Mechanistically, the biogenesis of apoVs is dependent on an apoptotic molecule-dependent distinctive mode, while different types of apoVs are characterized by their cellular origins and physiopathological contexts [38, 43, 44, 53]. Accumulating studies have revealed that apoVs play multiple important roles in animal development and in the maintenance of tissue and organ homeostasis [27, 56–58, 60, 61, 72, 77, 85, 107]. Subsequently, MSC-derived apoV infusion has been established as an effective biotherapy to cure dozens of diseases, including immune disorders, osteoporosis, skin injuries, tumors, and more [25].

Nevertheless, there are many unresolved issues and obstacles ahead that must be resolved for future clinical application. As a developing field, although apoV studies have been converging on a consistent conception of apoVs and their associated biological mechanisms, there are still problems concerning the unclear definition of apoVs in the growing literature due to the variety of tissues from which they have been derived and methods by which they have been separated (Table 2) [27, 38]. Furthermore, it is clear that the molecular and behavioral states of apoVs are highly age- or developmental stage-relative, which gives rise to functional discrepancies [53, 71]. Therefore, a portion of the studies on apoVs have inaccurately labeled the object of their study as apoBDs or apoptotic extracellular vesicles (apoEVs) [38, 53, 71]. Accordingly, establishing a uniform standard based on size, induction methods, and centrifugal force of isolation is highly warranted. On the other hand, apoptosis is exceptional as the most prominent mode of PCD, and it exerts unique and extensive physiopathological regulatory and therapeutic effects in vesicle-dependent ways; it remains to be investigated whether other cell death processes, including autophagy, necroptosis, and pyroptosis, may also generate vesicles that contribute to specific physiological and pathological modulatory activities [25]. Elucidating how products of cell death are involved in biological behaviors of organisms and therapeutic processes will shape our understanding of PCD and may uncover novel roles for this group of processes in tissue and organ homeostasis and disease progression. Last but not least, with the growing advances in techniques such as single vesicle analysis combined with transcriptomics, the characteristics and functional molecular signatures of apoVs in healthy and diseased conditions will progressively be revealed, producing novel knowledge of apoptosis and guiding the development of clinical therapeutics.
